# Root and Canal Morphology of Mandibular Molars in a Selected Iranian Population Using Cone-Beam Computed Tomography 

**DOI:** 10.22037/iej.2017.29

**Published:** 2017

**Authors:** Zahra Sadat Madani, Nika Mehraban, Ehsan Moudi, Ali Bijani

**Affiliations:** a *Department of Endodontics,Dental School, Babol University of Medical Sciences, Babol, Iran; *; b *General Dentist, Sari, Iran; *; c * Department of Oral and Maxillofacial Radiology, Dental School, Babol University of Medical Sciences, Babol, Iran; *; d *Non-Communicable Pediatric Diseases Research Center, Babol University of Medical Sciences, Babol, Iran*

**Keywords:** Cone-Beam Computed Tomography, C-Shaped Root Canals, Mandibular First Molar, Mandibular Second Molar, Root Canal Anatomy, Root Canal Morphology

## Abstract

**Introduction:**

The aim of this study was to evaluate the root canal morphology of mandibular first and second molars using cone-beam computed tomography (CBCT) in northern Iranian population and also to indicate the thinnest area around root canals.

**Methods and Materials:**

We evaluated CBCT images of 154 first molars and 147 second molars. By evaluating three axial, sagittal and coronal planes of each tooth we determined the number of root canals, prevalence of C-shaped Melton types, and prevalence of Vertucci configuration and inter orifice distance. Also the minimum wall thickness of root canals was determined by measuring buccal, lingual, distal and mesial wall thicknesses of each canal in levels with 2 mm intervals from apex to orifice.

**Results:**

Amongst 154 first mandibular molars, 149 (96.7%) had two roots, 3 (1.9%) had three roots and 2 (1.2%) had C-shaped root configuration. Of 147 second mandibular molars, 120 (81.6%) had two roots, 1 (0.6%) had three roots and 26 (17.6%) had C-shaped roots. There was no significant difference in the prevalence of Vertucci’s type between two genders. The most common configuration in mesial roots of first and second molars were type IV (57%-42.9%) and type II (31.5%-28%). Mesial and distal walls had the most frequency as the thinnest wall in all levels of root canals with mostly less than 1 mm thickness. In second molars the DB-DL inter orifice distance and in first molars the MB-ML distance were the minimum. MB-D in first molars had the maximum distance while ML-DL, MB-DB and ML-D had the same and maximum distance in second molars.

**Conclusion:**

Vertucci’s type IV and type I were the most prevalent configurations in mesial and distal roots of first and second mandibular molars and the thickness of thinnest area around the canals should be considered during endodontic treatments.

## Introduction

Today, by progression in modern dentistry, endodontic treatment has an effective and important role in teeth preservation [1]. Cleaning and shaping of the root canal system has great importance in root canal treatment (RCT) and reaching maximum success [2, 3] .

The result of successful endodontic treatment depends on knowledge, correct sight and awareness of root canal anatomy and careful, conservative and meticulously performed cleaning and shaping procedures. Lack of knowledge about root canal anatomy and its variations in configuration may lead to many root canal treatment failures such as perforation [3, 4].

The morphology of root canal systems varies greatly in different races and also among the different individuals within the same race, thus it is important to be aware of variations in tooth anatomy and its features in various racial groups because this knowledge can help clinician to locate and manage canals during root canal treatment [5]. A number of studies have evaluated the variations in number of roots and root canal types according to ethnicity and a literature search shows that comparatively few studies have evaluated the root anatomy of mandibular molars in ethnic populations by using cone-beam computed tomography (CBCT) [6].

**Figure 1:**
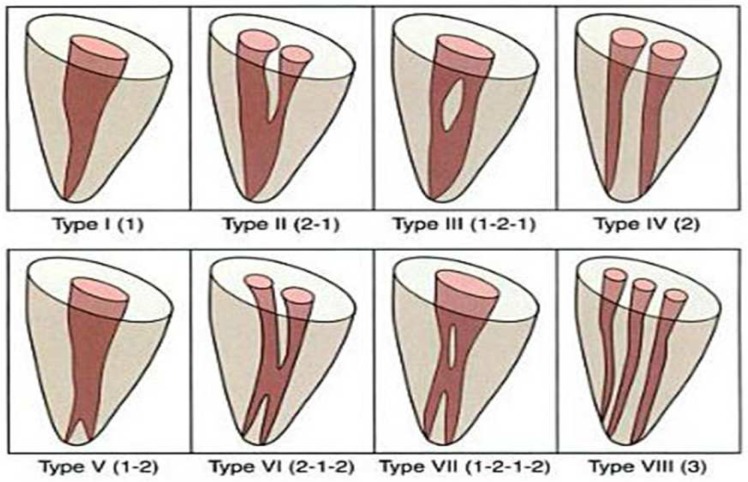
Vertucci root canal classification

Mandibular first molar is the first erupting posterior tooth witch is susceptible to decay or caries, thus in many cases it requires endodontic treatment. Most of the mandibular molars have one mesial and one distal root and there are two canals in the mesial root and one or two in the distal root in majority of them [3, 7]. Being aware of root canal morphology and the thickness of dentin around each canal specially in thinnest area enables the clinician to efficiently prevent procedural accidents and treatment failures [8]. 

The C-shaped root canal configuration was first categorized by Cooke and Cox in 1979 [9]. Because of the cross-sectional shape of the root and root canal anatomy they called C-shaped root canal [10]. C-shaped roots are formed by buccal or lingual fusion of the distal and mesial roots [11]. In these types of molars the orifice is ribbon-shaped with an arc of 180^º^ or more instead of the typical shape of the pulp chamber with three root canals. C-shaped root canals have a high prevalence in mandibular second molars. Melton *et al*. [12] made the first classification of C-shaped root canals and later Fan introduced an anatomic classification based on that [10].

Use of CBCT with its three-dimensional images has been widely used in endodontics in recent years and it has many advantages in comparison with routine two-dimensional radiographic techniques in evaluation of the root canal system [13]. 

The aim of this study was to evaluate three dimensional CBCT images of the root canal morphology of 301 fully erupted mandibular first and second molars in a selected northern Iranian population.

## Materials and Methods

This study evaluated the CBCT images of 110 patients referred to radiology clinic in Babol by other clinicians for CBCT imaging. So after their approval their CBCT images were used for this research. All CBCT images were performed by NewTom VG 9000 CBCT device (Quantitative Radiology SRL Co., Verona, Italy) with 7.09 × 6.30 FOV and 0.30 mm minimum isometric cubic voxel size. We evaluated fully erupted first and second mandibular molars without any endodontic treatment, resorption or root fracture. Samples included CBCT images of 154 first molars (from 92 female and 62 male patients) and 147 second molars (from 85 female and 62 male patients). CBCT cross-sections were 1 mm thick and were taken from 1mm segments of the canal from apical to coronal. Teeth were categorized by patients’ gender, mandible side (left or right), number of roots, number of root canals and root canal morphology (based on Vertucci’s classification) (Figure 1). 

To evaluate the CBCT images we used OnDemand 3D software (Cybermed Inc., Irvine, CA). Each tooth were evaluated in three axial, sagittal and coronal planes. We determined the number of roots in each tooth by evaluating the sagittal and coronal planes. In order to determine the number of root canals we evaluated axial plane from apex to orifice. The presence of C-shaped canal systems and their configurations were evaluated from the orifice to the apex in axial plane. C-shaped canal configuration was classified according to the classification by Melton *et al.* [12] and the modifications proposed by Fan *et al.* [14-16], the canals were classified as the following category: *Category I (C1)*-continuous C-shaped root canal from the orifice to the apex of the root; *Category II (C2)*-one main root canal and a smaller one; *Category III (C3)*-two or three root canals; *Category IV (C4)-*an oval or a round canal; *Category V (C5)*-no canal lumen or there is one close to the apex.

**Table1:** Configuration type of Vertucci and frequency for 154 first mandibular molars

**Root**	**Type I**	**Type II**	**Type III**	**Type IV**	**Type V**	**Type VI**
**Mesial**	11 (7.3%)	47 (31.5%)	3 (2%)	85 (57%)*	3 (2%)	0
**Distal**	119 (79.8%)*	16 (10.7%)	7 (4.6%)	5 (3.3%)	2 (1.3%)	0

**Table 2:** Configuration type of Vertucci and frequency for 147 second mandibular molars

**Root**	**Type I**	**Type II**	**Type III**	**Type IV**	**Type V**	**Type VI**
**Mesial**	22 (18.1%)	34 (28%)	7 (5.7%)	52 (42.9%)*	4 (3.3%)	1 (0.8%)
**Distal**	111 (91.7%)*	4 (3.3%)	1 (0.8%)	2 (1.6%)	2 (1.6%)	0

We measured inter-orifice distance with the ruler of the software from the edge of one canal to the other ones in the axial plane because only in this plane (axial) we could see the orifices and in other planes we may have superimpositions. The prevalence of Vertucci’s types (Figure 1) were determined by evaluating sagittal and axial planes. To determine the minimum wall thickness of root canals, buccal, lingual, distal and mesial wall thickness of each canals were measured in levels with 2 mm intervals from apex to orifice. Data was analyzed by SPSS software (version 20.0, SPSS, Chicago, IL, USA). We used X2, Fisher’s exact test and T test for statistical analysis and *P*-values less than 0.05 were considered as a significant difference. Independent sample T test was used for quantitative data with normal distribution, for comparing the left and right first and second mandibular inter-orifice distances.

## Results

Amongst 154 first mandibular molars 149 teeth (96.7%) had two roots, 3 (1.9%) had three roots and 2 (1.2%) had C-shaped configuration. Amongst 147 second mandibular molars, 120 teeth (81.6%) had two roots, 1 (0.6%) had three roots and 26 (17.6%) had C-shaped root. There was no significant difference in prevalence of Vertucci's types between left and right mandibular

molars (*P*>0.05). The most common canal configuration in mesial roots of first and second molars were type IV (57%-42.9%) and type II (31.5%-28%); but type I was the most common configuration in distal root (*P*<0.001) in both genders. Only one mesial root of second molar had type VI canal (Tables 1 and 2).

Of 301 mandibular molars, 28 teeth (9.3%) had C-shaped root canals and more specifically 26 were detected in second molars. Moreover, 22 of these C-shaped teeth were from female patients and 6 were found in males. The thinnest wall in level 1, 2 and 3 was LC (linguo central) and in level 4 was BC (bucco-mesial). The most common Melton type in level 1 was e, in level 2 was a and c, in level 3 was a, in level 4 was d and finally in level 5 was d (Tables 3 and 4).

Amongst mandibular molars in all levels, distal and mesial walls were the thinnest walls. In mesial roots of first molars from level 1 to level 5 we can see the increase in distal wall frequency as thinnest wall. In levels 1, 2 and 3 of the mesial root, the mean of thinnest thickness was less than 1 mm. In level 1 and 2 of distal root the mean of thinnest thickness of mesial and distal walls were less than 1 mm and in levels 3, 4 and 5. This mean for mesial wall is near 1 mm but for distal wall is near 1.5 mm. In levels 2, 3 and 4 of distal root, mesial wall had the most frequency as the thinnest wall.

In mesio-buccal canal at levels 1 and 6, mesial wall had the most frequency as the thinnest wall but in level 3 and 4 distal wall had more frequency. In mesiolingual canal distal wall had the most frequency as the thinnest wall in all levels except level 6. In both mesiolingual and mesiobuccal canals the thinnest mesial and distal walls had less than 1 mm mean of thickness in levels 1, 2 and 3.

**Table 3:** Frequency of the thinnest wall locations in each levels of the 28 C-shaped molars

**Location **	**Level 1**	**Level 2**	**Level 3**	**Level 4**	**Level 5**
**Bucco-central direction**	9 (32.1)	7 (25)	6 (21.4)	11 (44)	2 (33.3)
**Bucco-distal direction**	3 (10.7)	0 (0)	2 (7.1)	2 (8)	0 (0)
**Bucco-mesial direction**	1 (3.6)	3 (10.7)	3 (10.7)	3 (12)	2 (33.3)
**Lingo-central direction**	15 (53.6)	16 (57.1)	15 (53.6)	8 (32)	2 (33.3)
**Lingo-mesial direction**	0 (0)	2 (7.1)	2 (7.1)	1 (4)	0 (0)
**Total**	28	28	28	25	6

**Table 4:** Frequency of the 28 C-shaped molars configurations type in each levels

**Type**	**Level 1**	**Level 2**	**Level 3**	**Level 4**	**Level 5**
**Continuous C-shape canal**	5 (17.9)	7 (25)	9 (32.1)	7 (28)	0 (0)
**Semicolon-shape canal **	3 (10.7)	4 (14.3)	6 (21.4)	1 (4)	0 (0)
**Separated canal**	5 (17.9)	7 (25)	7 (25)	7 (28)	2 (33.3)
**Separated canal**	3 (10.7)	6 (21.4)	6 (21.4)	8 (32)	4 (66.6)
**One round or oval-shaped canal**	12 (42.9)	4 (14.3)	0 (0)	2 (8)	0 (0)
**Total**	28	28	28	25	6

**Figure 2:**
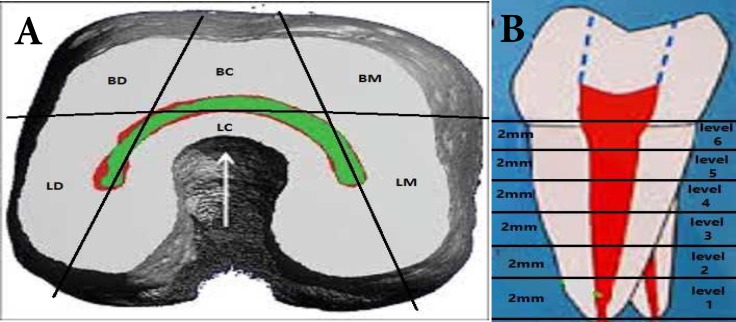
*A)* Measurement locations from apex to orifice of mandibular first and second molars with C-shaped canal systems; *B)* six sections were assessed and measured for the thinnest area (BM, bucco-central direction; LM, linguo-mesial direction; LC, linguo-central direction; LD, linguo-distal direction

Amongst mesial root of second mandibular molars in all levels distal wall had the most frequency as the thinnest wall and in levels 1 and 2 the mean of thickness in the thinnest mesial and distal wall was less than 1 mm. In distal root of second mandibular molars the mean of thickness in thinnest walls were less than 1 mm at level 1 and near 1 mm at level 2 but in level 3, 4 and 5 mesial walls had the mean of thickness near 1 mm as the thickness of distal walls were near 1.5 mm.

In mesiobuccal canal of second molars, mesial walls frequency as the thinnest wall were the most in level 1 but in the other levels distal wall had the most frequency. The mean of thickness for distal walls in level 1 and 2 were less than 1 mm, and were near 1 mm in the other levels. In mesiolingual canals distal wall had the most frequency as the thinnest wall in all levels with less than 1 mm mean of thickness in level 1, 2 and 3.

MB-DB has the maximum mean distance and DB-DL has the minimum mean distance in first and second mandibular molars. The mean distance of ML-DL (*P*=0.028) and MB-DB (*P*=0.017) in second mandibular molars and MB-ML (*P*=0.001) in first mandibular molars have significant different while the other distances has no significant different (Figure 2). 

## Discussion

Anatomy and morphology information of root canal system has important role in endodontic treatments. Missing canals and inappropriate instrumentation are the most reasons of endodontic treatment failures. 

Seven methods for the study of root canal system were evaluated by Neelakantan *et al. *[17]. These methods include CBCT, peripheral quantitative CT, spiral CT, plain (plain digital), contrast medium-enhanced digital radiographs, canal staining and tooth clearing techniques. They concluded that canal staining and tooth clearing technique are the gold standard methods and CBCT is as accurate and can be used also as an *in vitro* and *in vivo* method [17]. CBCT is a non-invasive method in comparison with the other techniques like clearing [18]. 

We chose mandibular molars because most of the dental patients need endodontic treatment for their mandibular molars. First mandibular molar is the first permanent tooth that erupts in oral cavity and C-shaped canals have high incidence in second mandibular molars. In our study 96.7% of first molars and 81.6% of second molars had two roots and we found three roots in 1.9% of first molars and 0.6% of second molars. The most common canal configuration in mesial roots of first and second molars were type IV (57%-42.9%) and type II (31.5%-28%) and the majority of distal roots were type I (79.8%-91.7%) like the most previous reports; but according to Manning [19], 3% of second mandibular molars had three roots and the most common configuration was type II in mesial roots and type I in distal roots [19]. In the study by Ahmad *et al.* [5] on Sudanese population, 78% of second mandibular molars had two roots which was less than our results Sperber and Moreau [20] and also Cruzon [21] reported a 3% prevalence for the third root in first mandibular molars. In the study by Shahi *et al.* [22] with clearing method, 1.44% of first molars had three roots which is close to our report. However, Zhang *et al.* [23] reported the high prevalence of third root in Asian population that was about 29%. In a CBCT study by Saberi *et al.* [8] in 49.5% of first molars mesial roots were type IV and 46.5% were type II but in our study type IV had higher prevalence and in the study by Wang *et al*. [24], the incidence of type IV was 94% that is higher than our study. Al Nazhan *et al.* [25] stated that in mesial roots of first mandibular molars, type II was the most common type. We didn’t find any mesial root with type VII and VIII in our study. Shahi *et al.* [22] also didn’t find any roots with these types but in many studies the prevalence of type VIII in mesial roots of first mandibular molars was 0.2-5% [22, 24, 26-28]. These variations are because of the influence of ethnicity and study methods.

C-shaped root canals are mostly found in second mandibular molars witch is more prevalent in Asian population rather than other ethnic groups [29]. In our study 17.6% of second mandibular molars had C-shaped root canals and the prevalence of C-shaped root canals in females were higher than the males that was similar to the study by Park [6]. Linguocentral walls were mostly the thinnest wall in these C-shaped molars with the nonlinear cubic curve pattern of decreasing in thickness. The most common type of Melton classification in our study were type III and IV in coronal and apical regions, respectively. According to Seo *et al.* [30], the most common type in coronal region was type I and in apical region was type III. They also reported the nonlinear cubic curve pattern of decreasing in thinnest wall thickness. We found that distal walls were mostly the thinnest walls in mesial canals of mandibular molars and had less than 1 mm thickness in apical levels of many cases. Thus, the possibility of perforation in distal walls are higher than the other walls that was similar to the report by Chang *et al.* [31] in which the distal walls of mesiolingual canals in first mandibular molars were thinner than mesial wall. 

## Conclusion

Vertucci’s type IV and type I were the most prevalent configurations in mesial and distal root of the first and second mandibular molars and the thickness of thinnest area around the canals should be considered during endodontic treatments..
